# Migration depth and residence time of juvenile salmonids in the forebays of hydropower dams prior to passage through turbines or juvenile bypass systems: implications for turbine-passage survival

**DOI:** 10.1093/conphys/cou064

**Published:** 2015-02-03

**Authors:** Xinya Li, Zhiqun D. Deng, Richard S. Brown, Tao Fu, Jayson J. Martinez, Geoffrey A. McMichael, John R. Skalski, Richard L. Townsend, Bradly A. Trumbo, Martin L. Ahmann, Jon F. Renholds

**Affiliations:** 1 Pacific Northwest National Laboratory, Hydrology Group, 3320 Innovation Boulevard, PO Box 999, MSIN K9-33, Richland, WA 99352, USA; 2 Pacific Northwest National Laboratory, Ecology Group, 3320 Innovation Boulevard, PO Box 999, MSIN K7-70, Richland, WA 99352, USA; 3 School of Aquatic and Fishery Sciences, University of Washington, 1325 Fourth Avenue, Suite 1820, Seattle, WA 98101, USA; 4 US Army Corps of Engineers, Walla Walla District, 201 N Third Avenue, Walla Walla, WA 99362, USA

**Keywords:** Acclimation depth, acoustic telemetry, juvenile salmonid, migration depth, three-dimensional tracking, turbine passage

## Abstract

Little is known about the three-dimensional depth distributions in rivers of individually marked fish that are in close proximity to hydropower facilities. Knowledge of the depth distributions of fish approaching dams can be used to understand how vulnerable fish are to injuries such as barotrauma as they pass through dams. To predict the possibility of barotrauma injury caused by pressure changes during turbine passage, it is necessary to understand fish behaviour relative to acclimation depth in dam forebays as they approach turbines. A guiding study was conducted using high-resolution three-dimensional tracking results of salmonids implanted with Juvenile Salmon Acoustic Telemetry System transmitters to investigate the depth distributions of subyearling and yearling Chinook salmon (*Oncorhynchus tshawytscha*) and juvenile steelhead (*Oncorhynchus mykiss*) passing two dams on the Snake River in Washington State. Multiple approaches were evaluated to describe the depth at which fish were acclimated, and statistical analyses were performed on large data sets extracted from ∼28 000 individually tagged fish during 2012 and 2013. Our study identified patterns of depth distributions of juvenile salmonids in forebays prior to passage through turbines or juvenile bypass systems. This research indicates that the median depth at which juvenile salmonids approached turbines ranged from 2.8 to 12.2 m, with the depths varying by species/life history, year, location (which dam) and diel period (between day and night). One of the most enlightening findings was the difference in dam passage associated with the diel period. The amount of time that turbine-passed fish spent in the immediate forebay prior to entering the powerhouse was much lower during the night than during the day. This research will allow scientists to understand turbine-passage survival better and enable them to assess more accurately the effects of dam passage on juvenile salmon survival.

## Abstract (precis)

We studied the depth and residence time of migrating juvenile salmonids in the forebays of hydropower dams before their passage through turbines or Juvenile Bypass Systems. This valuable information will increase our understanding of fish passage and help us identify possible management operations or better turbine designs.

## Introduction

Conservation and restoration of declining fish populations is a primary focus for many fisheries researchers and managers (MacIlwain, 1997; Wakefield, 2001; Miller, 2010). This focus has become increasingly important for river systems where hydropower facilities provide an obstacle for migratory fish passage or for areas where new hydro structures will be built, especially in areas where global human populations are expected to expand rapidly (Cooke *et al*., 2013a). Understanding telemetry-derived behaviour and linking it to their physiology (abilities to regulate buoyancy) can be key to conserving fish (Cooke *et al*., 2013b), in this case Pacific salmon, in systems where hydro facilities could negatively influence their populations. In the USA, many hydropower facilities are designed and operated to minimize impacts to fish passing both upstream and downstream. For example, at hydropower facilities in the lower Snake River, WA, USA, the US Army Corps of Engineers will be replacing ageing Kaplan turbines with new designs to minimize strike and shear as well as the rapid decreases in pressure to which fish are exposed (Trumbo *et al*., 2013).

Researchers have noted that, among juvenile salmonids, both large and rapid changes in pressure lead to barotrauma. They suggest that barotrauma is largely related to the expansion and rupturing of the swim bladder (Brown *et al*., 2012). Expansion of the gas within the swim bladder is in proportion to the decrease in pressure to which fish are exposed. As discussed by Van Heuvelen (1982), this expansion is described by Boyle's Law, which states that within a closed system (at a constant temperature) the volume of a gas is inversely proportional to the pressure acting on the volume. Thus, understanding how fish are injured by barotrauma is directly related to the ratio of the volume of gas within the swim bladder of a neutrally buoyant fish before it enters a turbine (often termed the acclimation pressure; Pflugrath *et al*., 2012) and the lowest pressure to which the fish are exposed during turbine passage (often termed the nadir pressure).

Identification of the low pressures to which fish are exposed during turbine passage is thus important for predicting barotrauma injury, but it is also critical to understand the physiology of the fish and know how much gas is in the swim bladder of fish as they approach the turbine. If two fish are exposed to the same low pressure during turbine passage, a fish with more gas in its swim bladder before passing the turbine will be more likely to receive barotrauma-related injuries (Tsvetkov *et al*., 1972), because the fish is exposed to a higher ratio of pressure change, the ratio being the pressure at which it was neutrally buoyant before entering the turbine divided by the lowest pressure the fish is exposed to while passing through the turbine. The greater the ratio of these two factors, the more the gas in the swim bladder expands and causes damage (Brown *et al*., 2014).

There is a lack of information about how much gas is in the swim bladders of fish before they enter turbines. One way of estimating the amount of gas would be to examine their behaviour and determine the depths that fish occupy while in the water column. It would be expected that fish—except for demersal fish such as sturgeon (*Suliformes*) and catfish (*Acipenseridae*)—would spend most of their time at a depth where they would expend the least energy and probably be neutrally buoyant. When neutrally buoyant, fish can minimize the amount of swimming that must be done to maintain their station and, thus, the amount of energy that is consumed (Moyle and Cech, 1982). When not neutrally buoyant, fish increase the amount they swim and the amount of energy used (Mehner, 2012). Diel period may also influence the vertical migration, which is a widespread phenomenon in aquatic organisms (Helfman, 1986; Clark and Levy, 1988; Scheuerell and Schindler, 2003). It may be helpful to examine the behaviour and determine whether or not a fish species occupies different depths in the water column in the forebay during day and night.

While many studies have described the vertical locations of fish within the water column in lakes or marine environs (including various swimming behaviours; Mehner, 2012), little high-resolution information exists for fish in rivers or in the forebays of dams immediately prior to passage through hydroturbines. In addition, until recently, technology has not been advanced enough to produce highly accurate locations of fish in the forebay of dams. Underwater acoustic tracking has been a common technology over the last two decades for studying and monitoring the movement and behaviour of aquatic animals in various applications. Since 2006, the Juvenile Salmon Acoustic Telemetry System (JSATS) has been used to estimate the survival and observe the seaward-migration behaviour of juvenile salmonids passing through eight large hydroelectric facilities operated by the US Army Corps of Engineers within the Federal Columbia River Power System en route to the Pacific Ocean (McMichael *et al*., 2010; Deng *et al*., 2011; Weiland *et al*., 2011). With the help of the more advanced techniques of underwater tracking to achieve high-resolution vertical locations of fish, one of the main objectives of our study was the development of an approach to evaluate the acclimation depth of seaward-migrating juvenile salmonids prior to the dam passage.

In this study, to provide insight into the vulnerability of juvenile salmonids to barotrauma for future evaluations of new turbine designs and operations, a large volume of data from a very large sample size of fish was investigated (Skalski *et al*., 2013a, 2013b, 2014). Data were extracted and analysed from over 28 000 individual fish implanted with JSATS acoustic transmitters encompassing three species/life-history types [yearling and subyearling Chinook salmon (*Oncorhynchus tshawytscha*) and steelhead (*Oncorhynchus mykiss*)]. These three-dimensional (3-D) data with sub-metre accuracy were compared across 2 years (2012 and 2013) in the forebays of two dams on Snake River, namely Little Goose Dam (LGS) and Lower Monumental Dam (LMN). Multiple methods were developed and compared to analyse 3-D behavioural tracks of acoustically tagged fish in the field in order to estimate the acclimation depth with a better precision prior to passage through dams. Highly accurate vertical depth distributions were identified and investigated for fish in the forebays prior to passage through turbines or juvenile bypass systems (JBSs) in relationship to the route they passed and the year and diel period (day vs. night).

## Materials and methods

### Study sites and cabled hydrophone array

**Figure 1:**
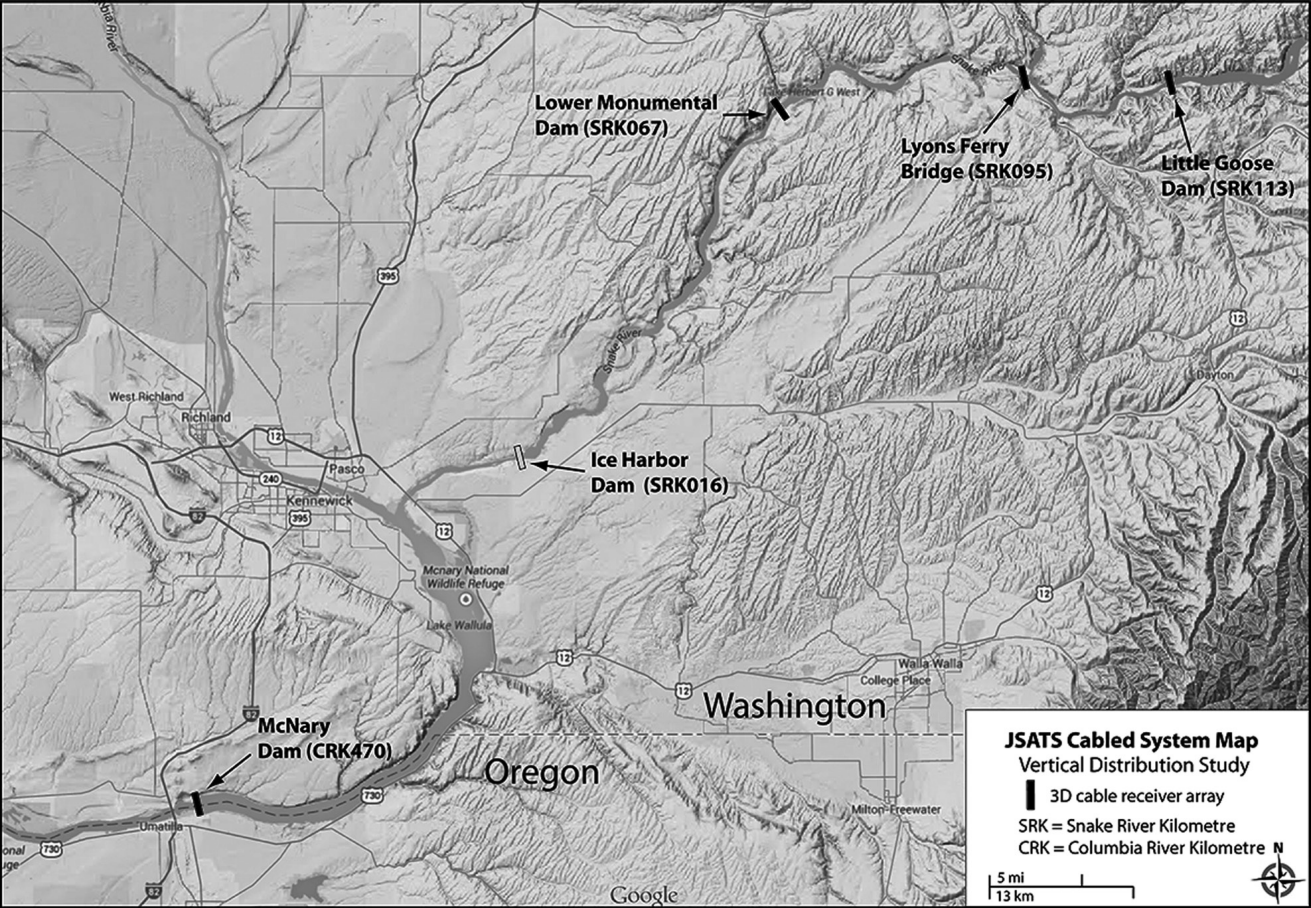
Locations of Juvenile Salmon Acoustic Telemetry System (JSATS)-cabled hydrophone arrays on the Snake River (Little Goose Dam and Lower Monumental Dam).

Little Goose Dam is located on the Snake River, 113 river kilometres upstream from the confluence with the main stem of the Columbia River, in south-central Washington State (Fig. 1). The dam is 809 m wide and 30.5 m tall and consists of a six-unit powerhouse, an eight-bay spillway, a navigation lock and an adult fish ladder. Lower Monumental Dam is located 46 river kilometres downstream of LGS (Fig. 1). The dam is 1155 m wide and 30.5 m tall and is similar in design to LGS, with two fish ladders and a juvenile fish facility.

**Figure 2:**
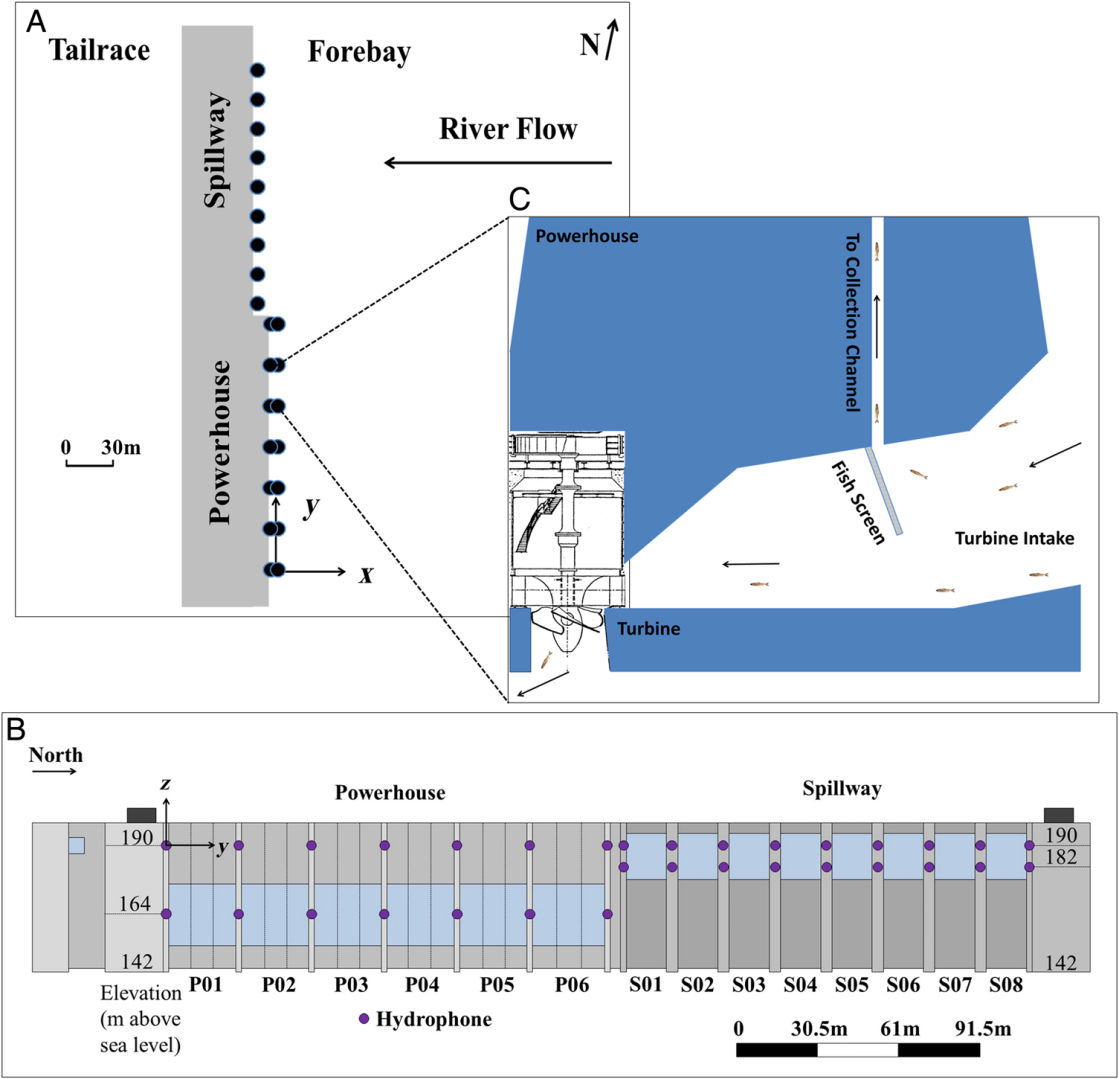
Deployment of JSATS hydrophones at Little Goose Dam on the Snake River. (**A**) Plan view. The black filled circles indicate the locations of the hydrophones. (**B**) Forebay view of locations of hydrophones deployed at two different elevations at each pier nose. The origin was at the normal pool surface level near the south end of the powerhouse. The *x* direction was perpendicular to the dam face looking into the forebay, while the *y* direction was parallel to the dam face. P01 indicates the first bay of the powerhouse and S01 is the first bay of the spillway. (**C**) Schematic cross-sectional view of turbine intake.

Fish implanted with JSATS acoustic transmitters were detected using JSATS-cabled hydrophone arrays (Weiland *et al*., 2011) deployed on the dam faces of LGS (Fig. 2A) and LMN. The cabled arrays were deployed using a design detailed by Skalski *et al*. (2014). Hydrophones were deployed on trolleys and lowered through pipes attached to the dam face. For each pier, two hydrophones were deployed at two depths at the powerhouse and spillway, respectively, to facilitate a 3-D tracking range throughout the water column (Fig. 2B). At LGS and LMN, all the shallow hydrophones were ∼3.5 m below the water surface, and the deep hydrophones installed at the powerhouses were 25 m deeper than shallow hydrophones, while the deep spillway (spill originating from a gate opening deep in the water column) hydrophones were 8–9 m deeper than shallow hydrophones.

The screened JBS guides fish away from turbines using a submerged screen installed in the turbine intakes (Harmon and Park, 1980). As fish travel with flow into the turbine intakes, the guidance screens guide the fish up through channels in the dam, routing them away from turbines. Fish swimming deeper into the turbine intake will skip the screen and pass through turbines to tailrace (Fig. 2C). The top of the turbine intake is ∼18 m deep below the water surface at both dams.

### Data collected and fish groups

An approximate maximum likelihood solver (Li *et al*., 2014) was applied to obtain reliable 3-D tracking results for several data sets (Table 1) including yearling and subyearling Chinook salmon and juvenile steelhead from 2012 at LGS and LMN (Skalski *et al*., 2013a, 2013b) and subyearling Chinook salmon from 2013 at LGS and LMN (Skalski *et al*., 2014).

**Table 1:** Cabled array locations (LGS, Little Goose Dam; LMN, Lower Monumental Dam in the Snake River, Washington), species or stock (CH0, subyearling Chinook salmon; CH1, yearling Chinook salmon; ST, steelhead), sample size (number of fish), size of fish, tag burdens and tag PRI of each field study season during 2012 and 2013

Year	Season	Cabled array location	Species/stock	Sample size (all passed)	Sample size (powerhouse passed)	Fork length range (mm)	Median (range) AT tag burden (%)	Tag PRI (s)
2012	Spring	LGS	Steelhead (ST)	1686	764	135−290	0.40 (0.16−1.58)	3.0
2012	Spring	LMN	Steelhead (ST)	3970	1357	132−311	0.40 (0.12−1.73)	3.0
2012	Spring	LGS	Yearling Chinook (CH1)	1661	611	95−191	1.40 (0.51−4.26)	3.0
2012	Spring	LMN	Yearling Chinook (CH1)	3898	837	95−238	1.40 (0.48−4.26)	3.0
2012	Summer	LGS	Subyearling Chinook (CH0)	2653	771	95−157	2.55 (0.86−4.61)	4.2
2012	Summer	LMN	Subyearling Chinook (CH0)	6204	1019	95−157	2.55 (0.80−4.61)	4.2
2013	Summer	LGS	Subyearling Chinook (CH0)	2428	599	95−143	2.81 (1.14−4.85)	4.2
2013	Summer	LMN	Subyearling Chinook (CH0)	5388	575	95−134	2.89 (1.34−4.75)	4.2

Abbreviations: AT, acoustic transmitter; PRI, pulse rate interval. Tag burden was calculated as weight of fish/weight of tag.

Several factors were examined among the tagged fish to allow better understanding of the depth distributions of these seaward-migrating salmonid species. One of these factors was whether diel period would influence the depth of the fish. Civil twilight time was used as the dividing point, thus allowing us to categorize the fish into day and night groups. A fish was placed in the ‘day’ group if it had more (>50%) day-time tracked points than night-time tracked points, or in the ‘night’ group if it had more night-time tracked points than day-time tracked points. A fish would be categorized to a ‘neutral’ group if it had equal numbers of tracked points during day and night. Although some fish may not have spent all of their time singularly in the day or night, most fish passed the dam quickly (the median residence time in the forebay was ∼15 minutes for all fish); thus, there was little opportunity for data to be mischaracterized as the incorrect diel period. In addition to diel period, data were examined to identify differences in depth distributions based on the species or life history of the fish, the passage route (including turbine, JBS, or both combined and termed ‘powerhouse’), which of the two dams and the year in which data were obtained.

### Fish acclimation depth analysis methods

Controlled field testing performed during 2012 and 2013 determined that sub-metre tracking accuracy of individual tagged fish locations was achievable within the forebays of dams when the fish were within 75 m of the horizontal distance to the dam face. Therefore, only 3-D tracking data within 75 m to the dam face in the forebay at the two dams (LGS and LMN) were included. This was done to exclude possible erroneous 3-D tracking points. After reliable 3-D tracking results and the associated tracking accuracy were obtained, five approaches were used to determine estimates of the depths where fish could be neutrally buoyant (i.e. acclimated). These five approaches were used only on powerhouse-passed fish within the forebay of dams. To avoid including biased depth data when a fish was not neutrally buoyant, an analysis was done to exclude the influence of the approach flow of the powerhouse.

This research advances five different approaches (Table 2) that can be used to examine vertical distribution data and estimate acclimation depths of seaward-migrating juvenile salmonids. A comparison of these approaches was conducted to determine which approach could be most applicable for further large-scale analyses of 3-D JSATS data. The goal of the approaches was to examine different ways for determining acclimation depth with a limited influence of the near-dam water velocities, but within locations where passage into the powerhouse was likely to occur relatively soon.

**Table 2:** Forebay near limits, forebay far limits, definitions of acclimation depth and scope of application in each of the fish acclimation depth analysis approaches for powerhouse-passed fish

Approach	Forebay near limit	Forebay far limit	Definition of acclimation depth	Scope of application
1	25 m	75 m	Median	Most straightforward; requires analysis on computational fluid dynamics or sensitivity analysis
2	25 m	75 m	Mode	Similar to Approach 1, but most probably represents the depth that fish achieve neutral buoyancy; requires sufficient data points for individual fish
3	Marked diving point	75 m	Mode	More complicated than Approaches 1 and 2; requires extensive analysis on the diving behaviour of individual fish
4	9 min before passage	75 m	Mode	Only effective when fish has similar searching and detouring time before passage
5	Marked acclimation segments (up to *x* = 75 m)		Mode	Most complicated; requires extensive analysis on a large number of fish for individual fish's depth detection history

Forebay near and far limits refer to areas that were close to or far from the dam face, respectively, to be included in the analysis. The definition of acclimation depth describes the statistical metric that was used for analysis.

The simplest way to exclude the period when fish were close to the turbine or spillway, and thus influenced by the flow velocities, was to set a horizontal distance limit. Tracked points within this distance limit to the dam face were excluded from the calculations of acclimation depth. This horizontal distance limit was determined according to a computational fluid dynamics study conducted for LMN (Stockstill *et al*., 2005). This study indicated that the surface spill and turbines can influence the flow as far as 22 m into the forebay in the water column. For a safer choice, 25 m was selected as the horizontal distance limit for Approaches 1 and 2, so there was little likelihood that the depth distributions of fish would be influenced by these higher velocities that could change fish behaviour. The median depth was the most straightforward choice for acclimation depth. Thus, this metric was used for Approach 1.

Another way to define acclimation depth was to find the depth at which each fish swam for the longest time, with the assumption that fish were most likely to be neutrally buoyant at this depth. This is called the ‘mode’ depth, which was used for Approach 2. The 3-D positions of fish were continuously tracked within the detection range of multiple hydrophones; in each segment of the trajectory, one position point was equal to the time interval of the tag pulse repetition interval (PRI; 3.0 or 4.2 s). The number of points was considered as a substitute for time. For the example, if the fish spent most of its time (largest number of tracked points) within a 1 m depth window from 6.3 to 7.3 m, the centre point of that 1 m window (i.e. 6.8 m) would be chosen as the acclimation depth for Approach 2.

**Figure 3:**
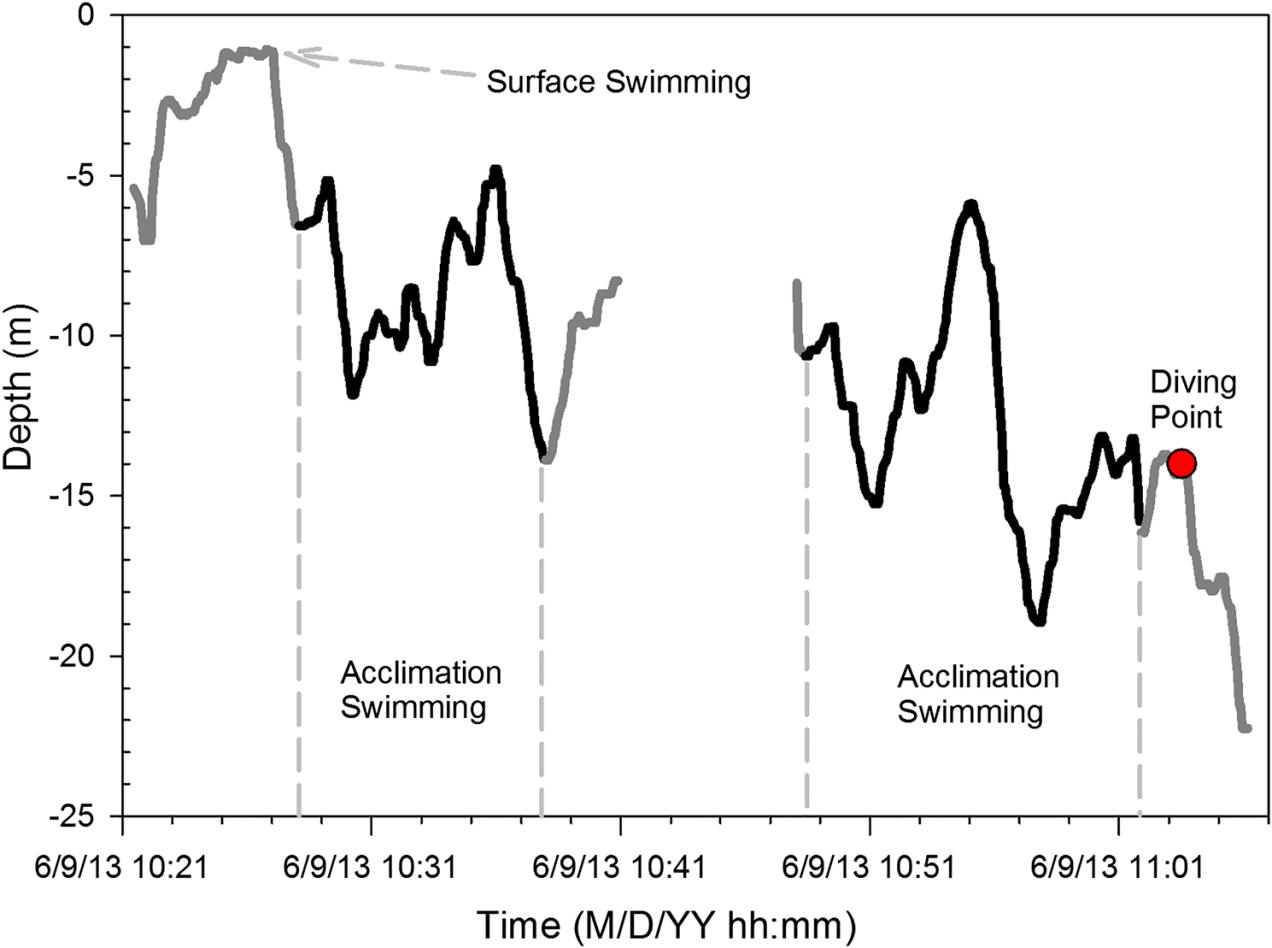
An example showing the start of diving (‘diving point’) of fish entering the powerhouse and the periods of time identified as ‘surface swimming’ and ‘acclimation swimming’ that were used for the acclimation depth calculation for Approach 5. There were periods when the fish moved outside of the detection range; this is indicated as a blank gap (representing about a 10 min period) in the middle of the figure.

A more specific way to exclude the period during which fish were influenced by the pulling velocities of the powerhouse was to find a point where the fish appeared to be diving into the powerhouse and then exclude data between that point and the point where the fish passed into the dam. This was chosen to be Approach 3. In addition to omitting data from the start of the dive until the passage through the dam, the acclimation depth was assigned using the depths that were occupied for the largest percentage of the time. Similar to Approach 2, the moving window technique was used as part of the criteria in Approach 3. The start of the diving point was defined as the last peak point in the often observed oscillation-like behaviour of a fish's trajectory before entering the powerhouse (Fig. 3). However, data were included only if they were higher in the water column than the top of the turbine intake (the opening for water inflow to the turbines). As shown in the example of a fish tracked at LGS (Fig. 3), the starting point for diving was marked with a red solid dot that is more than 5 m higher in the water column than the top of the turbine intake, which is 19.2 m deep. All the tracked points after the dive start point were excluded from calculations for this Approach 3.

Approach 4 used a time limit point for filtering data to be used. Data were used if they were associated with the time between the start of the diving point and the time of passage into the powerhouse. Examination of the diving point behavioural data indicated that 95% of the dives started within 9 min of the fish passing the dam; therefore, this time was used as a time-limiting criterion for Approach 4. Therefore, all data collected within 9 min before passage of the powerhouse were excluded from the moving window data for this method.

For Approach 5, fish swimming behaviour was again used to limit data that were assumed to represent acclimation. This included classifying data as ‘surface swimming’ or ‘acclimation swimming’. Surface swimming was defined as any data point when a fish was present within 1.5 m of the water surface. It was assumed that fish at these shallow depths would not be likely to have as much gas in their swim bladder as fish spending a considerable time deeper in the water column and on the approach to a powerhouse. Acclimation swimming was defined as the starting point of the first valley of oscillation-like swimming after the fish was detected at the surface (Fig. 3; within 1.5 m of the surface to account for possible detection distance error). The end-point of acclimation swimming was defined as the last valley point in oscillation-like swimming before the fish returned to the water surface (locations within 1.5 m of the surface) or when the fish left the reception range of detection gear. Between the starting and ending point of acclimation swimming, fish commonly oscillated around a certain depth that was assumed to be where the fish were acclimated or neutrally buoyant (and would be energetically optimal). The example track (Fig. 3) has two continuous track segments. The first segment illustrates one surface swimming period and one acclimation swimming period, while the second segment has only one acclimation swimming period. Only the acclimation tracked points in these two segments were included to evaluate acclimation depth. As in Approaches 2–4, the moving window mode technique depth data were used as part of the criteria in Approach 5.

### Statistical methods

After the acclimation depth was defined, statistical analyses were performed to conduct pairwise comparisons of the depth distributions between two different samples. This was done to investigate whether these two samples followed the same distribution at a significance level.

Using 3-D tracking information for each individual implanted fish, repeated observations of fish depths were estimated. For a fish group with *m* fish, let *x_ij_* be the depth estimate for the *j*th observation (*j* = 1 … *n_i_*) of the *i*th fish (*i* = 1 … *m*). For example, the acclimation depth of the *i*th fish, *d_i_*, was estimated by the mode of the *n_i_* observations from the *i*th fish. Here, the mode represents the depth at which the *i*th fish was most frequently observed, as follows:

$$\text{Mode}(x_{ij})=d_{i}\;i=1\ldots m$$

This value is an estimate whose precision depends on the sample size, *n_i_*.

For the large data sets available in this study, the variability of the tracked depth values from an individual fish and across fishes is not predictable. Without pre-knowledge for assumptions, the depth distribution of a fish group in this study is unknown and complicated by the possibility of insufficient sample size from the number of fish and the number of observations from a given fish. Hence, for each fish group, a two-stage bootstrapping procedure was used to construct a 95% confidence interval for the observed cumulative distribution function (CDF). The bootstrapping process has the following steps.

Step 1. Randomly sample with replacement of *m* fish from the original sample.
Step 2. For each fish randomly selected in Step 1, *n_i_* depth observations are randomly selected, with replacement for the original *n_i_* observations. For cases in which *n_i _*> 200, a random sample of size of *n* = 200 was used to reduce the processing time.
Step 3. From the *n_i_* bootstrap observations of the *i*th fish, a new depth distribution (i.e. mode) is estimated $\left(d_{i}^{\prime }\right)$.
Step 4. Using the *m* bootstrap values of $d_{i}^{\prime }(i=1,\ldots ,m)$, a new CDF was constructed.
Step 5. Steps 1–4 are repeated 1000 times, producing 1000 bootstrap estimates of the CDF.
Step 6. A 95% confidence interval for the original CDF was constructed by truncating the smallest 2.5% and the largest 2.5% of the bootstrap values of depth (*d_i_*) at each one percentile of the CDF.

For each comparison performed between two sample groups, a modified Kolmogorov–Smirnov test of equal distributions was performed (Conover, 1980), because the depth values were measured with error. Under the null hypothesis of equal distributions, the data from two alternative sample distributions were generated from a common distribution. As such, the distribution for the T-statistic can be empirically approximated by bootstrapping the pooled data from two alternative sample distributions. For example, one sample has *m*_1_ values of acclimation depth; the other, *m*_2_ values. The procedure is described as follows.

Step 1. Pool the *m*_1_ and *m*_2_ observations into a common CDF for a replication in the bootstrapping procedure.
Step 2. Randomly select *m*_1_ observations for the first sample and *m*_2_ observations for the second sample with replacement.
Step 3. Construct the prospective CDFs for the two samples and calculate the maximal vertical distance, *D*.
Step 4. Repeat Steps 1–3 1000 times and construct an empirical distribution for the test statistic *D* under the null hypothesis.
Step 5. Determine the placement of the actual T-test statistic within the empirical distribution. The *P*-value was calculated as the number of equal or more extreme values in the tail × 2/1000.

The *P*-value is the probability that the observed difference would occur, given that the null hypothesis (the vertical distributions are the same) is true. A *P*-value ≥ 0.05 was adopted as the criterion that the two sample groups were not significantly different. The *P*-value tends to be smaller as sample size increases, unless the null hypothesis that the two sample groups are from the same distribution is true. Hence, low sample size can lead to large confidence intervals on the estimated distribution. Data within several tables are referred to as the median depth and also the most common depth. The most common depth was characterized as the maximal depth at which the majority of fish passed. It was the specific depth at which 75% of the fish resided or shallower than that value. In other words, only 25% of fish were acclimated at a depth deeper than that value. This break-point of 75% was used because box plots typically include the median within a box that illustrates where 25 and 75% of the data are located. Although this allows for a way to visualize the data, the original intent of this research was to identify multiple, highly accurate vertical depth distributions.

## Results

### Approaches to estimating acclimation depth

Given that the primary purpose of this research was to estimate the depth where juvenile salmonids may be neutrally buoyant (termed ‘acclimation depth’ for this analysis) prior to turbine passage, their 3-D locations were treated in several different ways to see whether there was a difference among the approaches. Among the five different approaches, there were differences in the estimates of acclimation depth (Table 3); however, all of the approaches but one led to similar results. Among most approaches (except for Approach 4), fish passing via the turbines were detected deeper in the water column [most (75%) had acclimation depths ≤19.2 m (Table 3)] than those that passed via the JBSs [most (75%) had acclimation depths ≤13.6 m].

**Table 3:** Median depth at which subyearling Chinook salmon were detected within the forebay of Little Goose Dam (in the Snake River, Washington) during 2013

Approach	Turbine passed			JBS passed		
	*n*	Median depth (m)	Most common depth (75%; m)	*n*	Median depth (m)	Most common depth (75%; m)
1	129	13.3	19.2	470	10.0	12.9
2	129	12.2	18.4	470	9.5	13.0
3	120	11.7	17.0	465	9.9	13.2
4	128	16.8	24.0	472	12.9	15.4
5	120	13.1	17.5	453	11.0	13.6

Depths are shown for five different approaches to estimating the acclimation depth of the fish. All of the comparisons of depth distributions between turbine- and JBS-passed fish were significantly different (*P* < 0.001). The median depth is shown, as well as the depth at which 75% of the fish were found or were shallower than. Abbreviations: JBS, juvenile bypass system; *n*, sample size, number of fish.

The acclimation estimates for turbine-passed and JBS-passed fish conducted using Approach 4 led to notably different, deeper estimates than the other four approaches. For example, among fish passing turbines, most approaches (except for Approach 4, which had median depths of 16.8 m) had median depths from 11.7 to 13.3 m; a range of only 1.6 m. Among all approaches except Approach 4, the majority (75% of fish) of turbine-passed fish were detected at 17.0–19.2 m or less (a range of only 2.2 m). However, among turbine-passed fish examined using Approach 4, both the median (16.8 m) and the majority of their depths (75% of fish; 24.0 m or less) were far outside the range of the other four approaches. A similar pattern was present among JBS-passed fish. Data produced using Approach 4 were considered to be outliers among all of the approaches, considering that distance limit was a more reasonable criterion than time limit, excluding influence from the powerhouse.

Several factors were used to determine which approach may provide the most relevant and reasonable estimates of acclimation depth. Approach 2 provided values that were centrally located among the four approaches (excluding Approach 4). In addition, Approach 2 uses a much less complex way to calculate acclimation depth than Approaches 3 and 5, but it is similar in complexity to Approach 1. Approaches 3 and 5 define the acclimation depth according to the passage behaviour of individual fish, which required extensive analysis of each fish's depth detection data history, conducted on a large number of fish, to be able to calculate criteria. Thus, the more simplistic Approach 2 was used to present the rest of the acclimation information because of its simplicity and results that were fairly centrally located among all methods except for Approach 4.

### Differences between turbine-passed and juvenile bypass system-passed fish

Although this research was largely conducted to identify distributions of fish to help estimate survival during turbine passage, the data can provide valuable insight about general fish behaviour near dams and how this behaviour may differ depending on the type of fish, the different dams, the diel period or the year and conditions in which fish passed. Given that powerhouses can be run either with or without screens intended to guide fish into JBSs, estimates of vertical distributions of fish entering through both the turbines and the JBSs have value, as do comparisons of the differences between the two possible routes.

Among the multiple comparisons of fish passing through the powerhouse, most fish passing through turbines had a deeper vertical distribution than those passing through JBSs. During 2013, when only subyearling Chinook salmon were studied, fish entering the turbines had significantly (*P* < 0.05) deeper estimated vertical depth distributions, with medians of 12.2 m at LGS and 11.0 m at LMN, compared with those that entered through the JBS (medians of 9.5 m at LGS and 8.9 m at LMN; Table 4). In addition, most (75%) of the fish passing through turbines resided deeper (≤18.4 m at LGS and 17.0 m at LMN) in the forebay prior to passing than those passing via the JBS (≤13.0 m at LGS and 13.8 m at LMN).

**Table 4:** Median depth at which three types of juvenile salmonids (CH0, subyearling Chinook salmon; CH1, yearling Chinook salmon; ST, steelhead) were detected within the forebay of LGS and LMN dams (in the Snake River, Washington) during 2012 and 2013

Year	Fish species	Location	*P*-Value	Turbine-passed fish			JBS-passed fish		
				*n*	Median depth (m)	Most common depth (75%; m)	*n*	Median depth (m)	Most common depth (75%; m)
2012	CH1	LGS	0.132	66	4.6	9.1	545	3.0	6.8
2012	ST	LGS	0.016	34	7.5	24.9	730	4.9	8.4
2012	CH0	LGS	<0.001	139	8.0	15.7	632	3.9	9.0
2012	CH1	LMN	0.012	206	2.8	6.4	631	2.8	5.2
2012	ST	LMN	<0.001	137	7.0	13.1	1220	6.1	9.3
2012	CH0	LMN	<0.001	470	7.1	15.6	549	4.7	10.1
2013	CH0	LGS	<0.001	129	12.2	18.4	470	9.5	13.0
2013	CH0	LMN	<0.001	256	11.0	17.0	319	8.9	13.8

The median depth is shown, as well as the depth at which 75% of the fish were found or were shallower than. The probability (*P*) of the difference, if there was a common vertical distribution between turbine-passed and JBS-passed fish, is shown.

**Figure 4:**
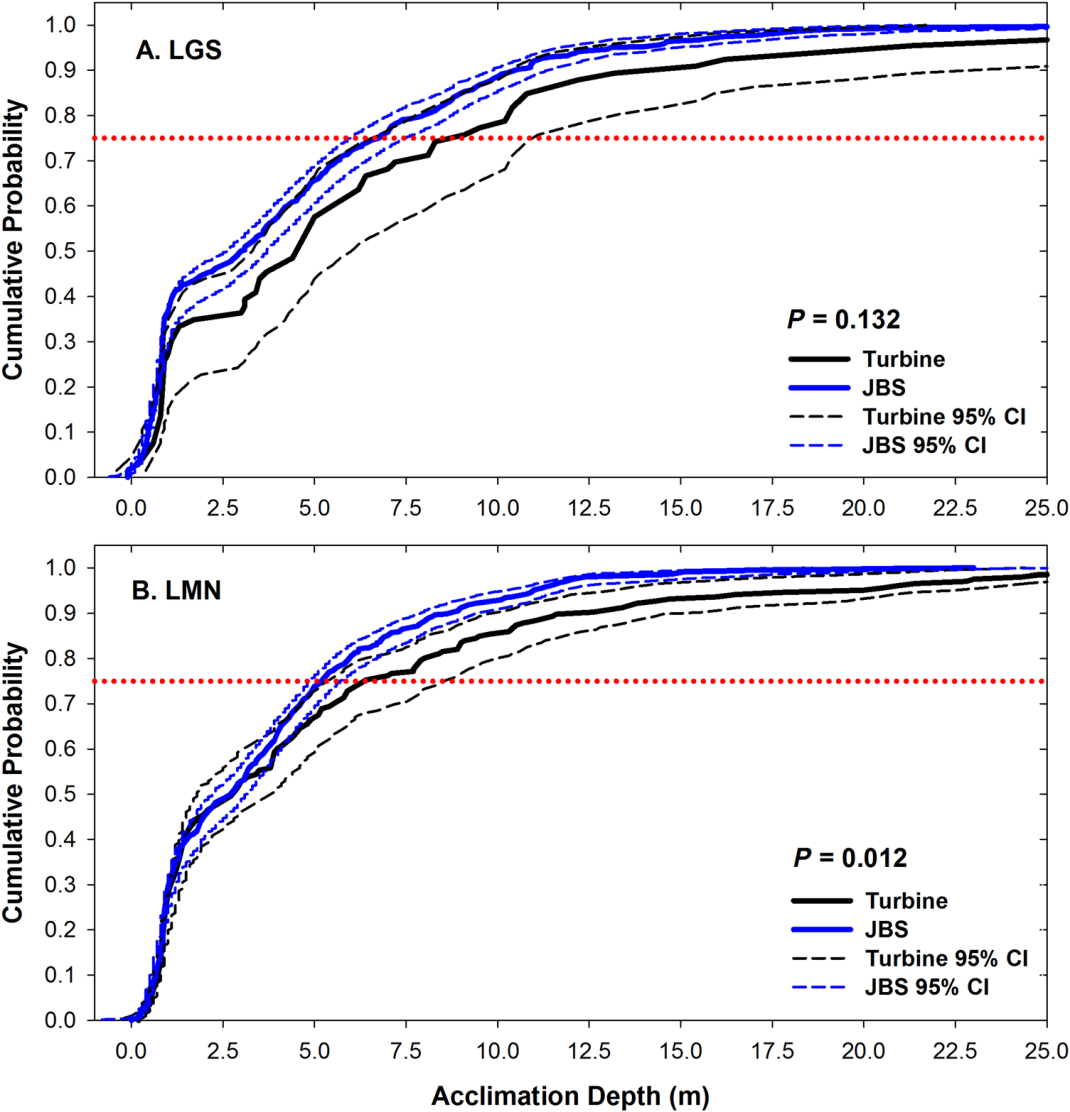
(**A**) A comparison of depth distributions of yearling Chinook salmon implanted with acoustic transmitters during 2012 passing Little Goose Dam (LGS) through either the turbines or the juvenile bypass system (JBS). There was no significant (*P* ≥ 0.05) difference in the depth distributions of JBS- and turbine-passed fish. (**B**) A comparison of depth distributions of yearling Chinook salmon implanted with acoustic transmitters during 2012 passing Lower Monumental Dam (LMN) through either the turbines or the JBS. There was a significant difference (*P* < 0.05) in the depth distribution of JBS and turbine-passed fish. The 95% confidence intervals (CIs) for each depth distribution are included. The dotted horizontal red line indicates that 75% of the fish were residing at or shallower than the corresponding depth.

Among fish studied during 2012, all of the vertical distributions of turbine-passed fish (with median depths of 2.8–8.0 m) were significantly (*P* < 0.05) deeper than those of JBS-passed fish (with median depths of 2.8–6.1 m) except for one (Table 4). There was not a significant difference (*P* = 0.132) in vertical distributions between yearling Chinook salmon passing through the turbines and the JBS at Little Goose Dam (Fig. 4A). Although the median depth of passage was identical between turbine-passed and JBS-passed yearling Chinook salmon at LMN (2.8 m; Table 4), there was a statistical difference (*P* = 0.012; Fig. 4B).

**Figure 5:**
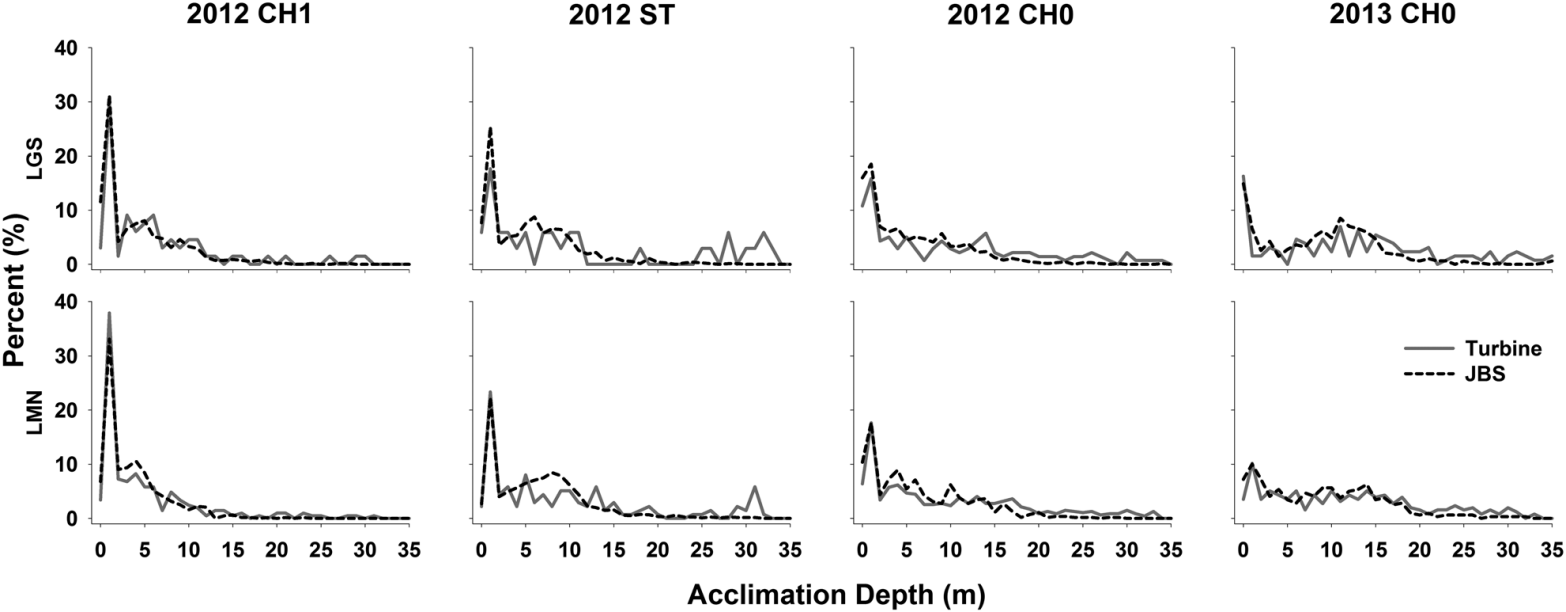
Depth–percentage curves of eight pairwise comparisons between turbine-passed (grey continuous line) and JBS-passed fish (black dashed line) listed in Table 4, where three types of juvenile salmonids (CH0, subyearling Chinook salmon; CH1, yearling Chinook salmon; ST, steelhead) were detected within the forebay of Little Goose (LGS) and Lower Monumental (LMN) dams (in the Snake River, Washington) during 2012 and 2013. Water surface is at 0 m.

By observing the depth–percentage curves (Fig. 5), it is clear that, for yearling Chinook salmon, 30–40% of fish resided near the water surface in the forebay for both turbine-passed and JBS-passed fish, while only a small percentage of fish resided at a deep depth (>15 m). For turbine-passed steelhead and subyearling Chinook salmon, a higher percentage of turbine-passed fish appeared to be acclimated at a depth deeper than 15 m relative to JBS-passed fish.

### Differences between years

Subyearling Chinook salmon (the only fish type tagged both during 2012 and 2013) that entered turbines during 2013 had significantly (*P* < 0.001) deeper depth distributions than those that entered during 2012 (Table 5). This was the case for the fish passing at both LGS and LMN. Most of the fish (75%) that passed into turbines during 2012 were 15.7 and 15.6 m deep or less at LGS and LMN, respectively. However, during 2013, most were 18.4 m deep at LGS and 17.0 m deep at LMN. In addition, the median depths of turbine-passed fish were much shallower during 2012 (8.0 m at LGS and 7.1 m at LMN) than during 2013 (12.2 m at LGS and 11.0 m at LMN).

**Table 5:** Median depth at which subyearling Chinook salmon were detected within the forebay of LGS and LMN dams (in the Snake River, Washington) during 2012 and 2013 prior to passage through either the turbines or JBS

Passage route	Location	2012			2013		
		*n*	Median depth (m)	Most common depth (75%; m)	*n*	Median depth (m)	Most common depth (75%; m)
Turbine	LGS	139	8.0	15.7	129	12.2	18.4
Turbine	LMN	470	7.1	15.6	256	11.0	17.0
JBS	LGS	632	3.9	9.0	470	9.5	13.0
JBS	LMN	549	4.7	10.1	319	8.9	13.8

The median depth is shown, as well as the depth at which 75% of the fish were found or were shallower than. All of the comparisons of depth distributions between 2012 and 2013 fish were significantly different (*P *<* *0.001).

Similar to turbine-passed fish, the depth distributions of JBS-passed fish were significantly (*P* < 0.001) deeper during 2013 than during 2012. This was the case for fish passing at both LGS and LMN. Most of the fish (75%) that passed into turbines during 2012 were 9.0 m deep at LGS and 10.1 m deep at LMN, while most were 13.0–13.8 m deep during 2013 (at LGS and LMN, respectively). The median depths of JBS-passed fish were much deeper during 2013 (9.5 m at LGS and 8.5 m at LMN) than during 2012 (3.9 m at LGS and 4.7 m at LMN).

### Differences between species and life history

Among turbine-passed fish, yearling Chinook salmon were significantly (*P* < 0.05) shallower than both subyearling Chinook salmon and steelhead at both LGS and LMN (Table 6). The median acclimation depths for yearling Chinook salmon were 2.9 and 4.2 m shallower than for steelhead, and 3.4 and 4.3 m shallower than for subyearling Chinook salmon at LGS and LMN, respectively. However, there were no significant differences (*P *≥* *0.05) between the depth distributions of subyearling Chinook salmon and steelhead; their median depths were <1 m different at both LGS and LMN. However, there was high variability among the acclimation depths of steelhead passing LGS, with 25% of fish found 24.9 m or deeper in the water column, while the median acclimation depth was only 7.5 m.

**Table 6:** Comparison of acclimation depths of three groups of juvenile salmonids passing through either turbines or JBSs at LGS or LMN dams, in the Snake River in Washington State

Passage route	*P*-Value	Species A				Species B			
		*n*		Median depth (m)	Most common depth (75%; m)	*n*		Median depth (m)	Most common depth (75%; m)
Turbine/LGS	0.044	66	CH1	4.6	9.1	34	ST	7.5	24.9
Turbine/LGS	<0.001	66	CH1	4.6	9.1	139	CH0	8.0	15.7
Turbine/LGS	0.436	34	ST	7.5	24.9	139	CH0	8.0	15.7
Turbine/LMN	<0.001	206	CH1	2.8	6.4	137	ST	7.0	13.1
Turbine/LMN	<0.001	206	CH1	2.8	6.4	470	CH0	7.1	15.6
Turbine/LMN	0.25	137	ST	7.0	13.1	470	CH0	7.1	15.6
JBS/LGS	<0.001	545	CH1	3.0	6.8	730	ST	4.9	8.4
JBS/LGS	<0.001	545	CH1	3.0	6.8	632	CH0	3.9	9.0
JBS/LGS	<0.001	730	ST	4.9	8.4	632	CH0	3.9	9.0
JBS/LMN	<0.001	631	CH1	2.8	5.2	1220	ST	6.1	9.3
JBS/LMN	<0.001	631	CH1	2.8	5.2	549	CH0	4.7	10.1
JBS/LMN	<0.001	1220	ST	6.1	9.3	549	CH0	4.7	10.1

The median depth is shown, as well as the depth at which 75% of the fish were found or were shallower than. The probability (*P*) of the difference, if there was a common vertical distribution between fish types, marked as species A or species B for each comparison, is shown. All of the comparisons among species were conducted using data only from 2012 because only subyearling Chinook salmon were studied during 2013.

Similar to turbine-passed fish, JBS-passed yearling Chinook salmon depth distributions were significantly (*P* < 0.05) shallower than for both subyearling Chinook salmon and steelhead at both LGS and LMN. The median acclimation depths of yearling Chinook salmon were 0.9–3.3 m shallower than for steelhead and subyearling Chinook salmon at both dams. While depth distributions were significantly different (*P* < 0.05) between steelhead and subyearling Chinook salmon, there were similarities between them. This can be seen by the fact that the median values were similar, with subyearling Chinook salmon having median depths of 3.9 m at LGS and 4.7 m at LMN, while steelhead had median depths slightly deeper, at 4.9 and 6.1 m at the same locations. While steelhead had only slightly deeper median depths than subyearling Chinook salmon, the opposite was true when examining a larger portion of the depth distributions. For example, 75% of subyearling Chinook salmon were 9.0 and 10.1 m deep or less at LGS and LMN, respectively, while most (75%) steelhead had acclimation depths of 8.4 and 9.3 m at the same locations.

### Differences by location

Fish passing via turbines generally had deeper depth distributions at LGS than at LMN, with medians 0.5–1.8 m deeper at LGS (Table 7). However, this relationship was not significant (*P* ≥ 0.05) for either steelhead or subyearling Chinook salmon during 2012, with differences in medians of only 0.5 and 0.9 m, respectively. The differences in acclimation depth between the two dams was significantly (*P* < 0.05) larger among the yearling Chinook salmon tracked during 2012 and the subyearling Chinook salmon tracked during 2013, where differences in medians were 1.8 and 1.2 m, respectively.

**Table 7:** Median depth at which three types of juvenile salmonids were detected within the forebay of Little Goose and Lower Monumental dams (in the Snake River, Washington) during 2012 and 2013

Year	Fish species	Passage route	*P*-Value	Little Goose Dam			Lower Monumental Dam		
				*n*	Median depth (m)	Most common depth (75%; m)	*n*	Median depth (m)	Most common depth (75%; m)
2012	CH1	Turbine	0.026	66	4.6	9.1	206	2.8	6.4
2012	ST	Turbine	0.706	34	7.5	24.9	137	7.0	13.1
2012	CH0	Turbine	0.694	139	8.0	15.7	470	7.1	15.6
2013	CH0	Turbine	0.012	129	12.2	18.4	256	11.0	17.0
2012	CH1	JBS	<0.001	545	3.0	6.8	631	2.8	5.2
2012	ST	JBS	<0.001	730	4.9	8.4	1220	6.1	9.3
2012	CH0	JBS	<0.001	632	3.9	9.0	549	4.7	10.1
2013	CH0	JBS	0.024	470	9.5	13.0	319	8.9	13.8

The median depth is shown, as well as the depth at which 75% of the fish were found or were shallower than. The probability (*P*) of the difference, if there was a common vertical distribution between the two different dams, is also shown.

Juvenile bypass system-passed steelhead and subyearling Chinook salmon studied during 2012 had significantly (*P* < 0.05) deeper depth distributions at LMN than at LGS (Table 7). This is reflected in the median depths being 0.8 and 1.2 m deeper among subyearling Chinook salmon and steelhead, respectively. Yearling Chinook salmon passing through JBSs had slightly (median depth being only 0.2 m deeper) although significantly (*P* < 0.001) deeper depth distributions at LGS than at LMN. Juvenile bypass system-passed subyearling Chinook salmon studied in 2013 had significantly different depth distributions between LGS and LMN, with slightly deeper (0.6 m deeper) median depths at LGS than at LMN. However, the opposite was true when examining a larger portion of the depth distributions. For example, 75% of the fish passing LMN were 13.8 m deep or less, while those passing LGS were 13.0 m deep or less.

### Diel differences

**Figure 6:**
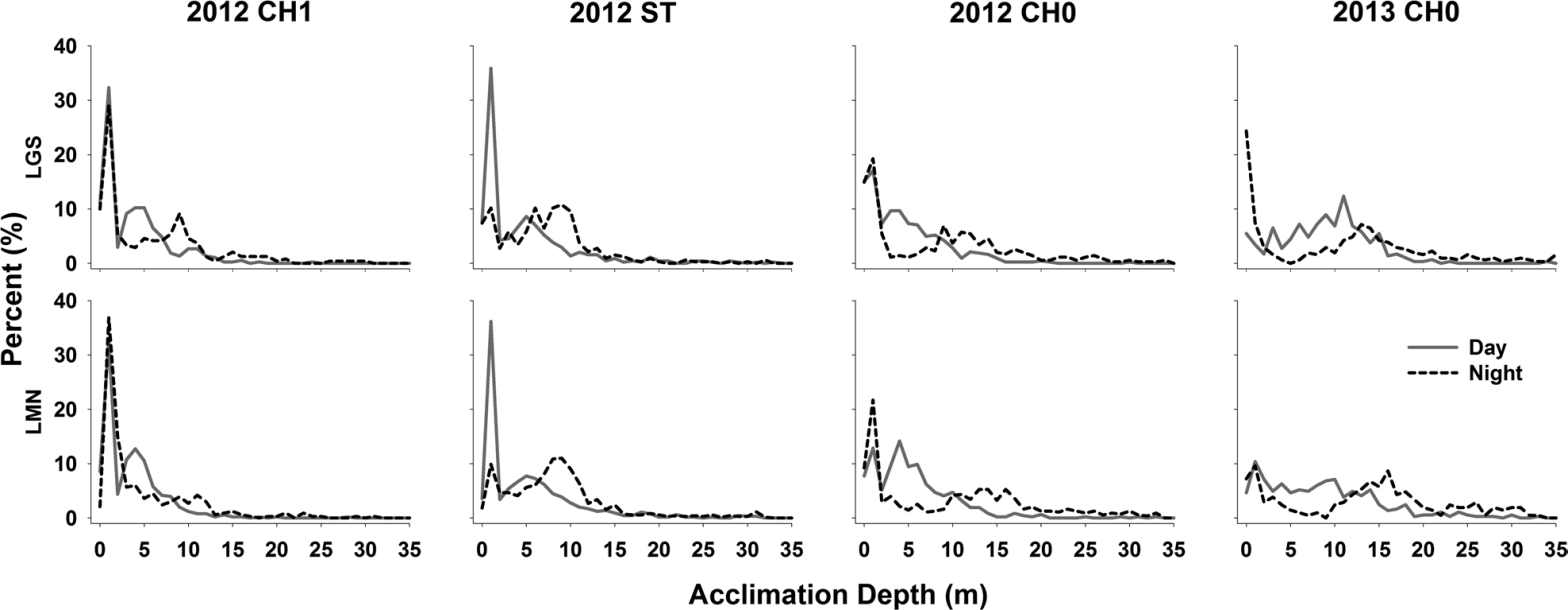
Depth–percentage curves of eight pairwise comparisons between day-passed (grey continuous line) and night-passed fish (black dashed line) listed in Table 8, where three types of juvenile salmonids (CH0, subyearling Chinook salmon; CH1, yearling Chinook salmon; ST, steelhead) were detected within the forebay of Little Goose (LGS) and Lower Monumental (LMN) dams (in the Snake River, Washington) during 2012 and 2013. Water surface is at 0 m.

Powerhouse-passed fish (JBS-passed and turbine-passed fish combined) generally were deeper in the water column during the night than during the day (Table 8). Among the three fish types, subyearling Chinook salmon had some of the largest differences (all being significant; *P* < 0.001) in depth distributions between day and night. Their median acclimation depth was 2.2–6.4 m deeper during the night than during the day (Table 8). Steelhead had median acclimation depths 3.8–4.2 m deeper (and significantly different; *P* < 0.001) during the night than during the day. Among the three fish types, yearling Chinook salmon had the least amount of difference between the depth distributions during day and night. Median acclimation depth for yearling Chinook salmon was 1.2 m deeper (and significantly different; *P* < 0.001) during the night than during the day at LGS, while median acclimation depth was 0.9 m shallower at night than during the day at LMN. However, despite median acclimation depths of yearling Chinook salmon being deeper during the day at LMN, most (75%) of the fish that passed at night had acclimation depths ≤7.0 m, while most of the fish passing during the day were shallower (≤5.0 m). For all three species in 2012 and 2013, there were higher percentages of fish acclimated at depths >10 m for night-passed fish than for day-passed fish (Fig. 6).

**Table 8:** Median depth during day and night at which three types of juvenile salmonids were detected within the forebay of LGS and LMN dams (in the Snake River, Washington) during 2012 and 2013

Year	Fish species	Location	Passed during day			Passed during night		
			*n*	Median depth (m)	Most common depth (75%; m)	*n*	Median depth (m)	Most common depth (75%; m)
2012	CH1	LGS	371	3.1	5.4	240	4.3	9.3
2012	ST	LGS	440	3.3	6.6	324	7.1	9.7
2012	CH0	LGS	423	3.7	7.0	348	8.5	13.3
2012	CH1	LMN	503	3.0	5.0	334	2.1	7.0
2012	ST	LMN	647	3.8	7.2	709	8.0	10.4
2012	CH0	LMN	466	4.6	7.5	553	10.1	15.8
2013	CH0	LGS	291	9.2	12.0	308	11.4	16.3
2013	CH0	LMN	366	8.0	12.0	208	14.4	18.5

The median depth is shown, as well as the depth at which 75% of the fish were found or were shallower than. All of the comparisons of depth distributions between day- and night-passed fish were significantly different (*P *<* *0.001).

The amount of time it took turbine-passed fish to pass the forebay receiving array and enter the powerhouse was much less during the night than during the day (Table 9). Among the fish passing the main study zone (between 25 and 75 m from the face of the dam), the range in time to pass was 8.1–246.7 min during the day and 3.3–11.2 min during the night. The mean of all of the passage time estimates for this area was 94.1 min during the day but only 5.5 min during the night.

**Table 9:** The median residence time (in minutes) during day and night in the forebay where three types of juvenile salmonids were detected at LGS and LMN dams during 2012 and 2013 before passing turbine or JBS

Year	Location	Species	Diel period	Median residence time (min) in forebay before passing turbine			Median residence time (min) in forebay before passing JBS		
				*n*	75−25 m	25 m–dam face	*n*	75−25 m	25 m–dam face
2012	LGS	CH1	Day	33	90.4	110.1	338	119.1	116.9
2012	LGS	CH1	Night	33	4.1	1.7	207	5.0	3.7
2012	LGS	ST	Day	20	35.6	30.7	420	81.5	60.4
2012	LGS	ST	Night	14	4.0	3.2	310	5.6	8.4
2012	LGS	CH0	Day	60	97.2	82.9	363	148.9	155.1
2012	LGS	CH0	Night	79	6.1	1.3	269	6.4	3.3
2013	LGS	CH0	Day	41	246.7	47.1	250	39.2	41.7
2013	LGS	CH0	Night	88	11.2	1.8	220	20.8	8.1
2012	LMN	CH1	Day	116	11.3	50.3	387	28.5	81.2
2012	LMN	CH1	Night	90	3.3	5.3	244	3.1	4.4
2012	LMN	ST	Day	67	8.1	26.6	580	30.8	25.8
2012	LMN	ST	Night	70	3.3	4.7	639	4.2	7.9
2012	LMN	CH0	Day	198	231.1	180.7	268	130.5	118.0
2012	LMN	CH0	Night	272	4.9	2.0	281	5.1	2.8
2013	LMN	CH0	Day	148	32.7	35.2	218	31.8	41.7
2013	LMN	CH0	Night	107	6.8	2.5	101	7.1	3.5

The median residence times are shown by two separated values: ‘75–25 m’ indicates the area in the forebay from the horizontal distance of 75 m away to 25 m away from the dam face; ‘25 m–dam face’ indicates the area in the forebay from the horizontal distance of 25 m away to the dam face.

The pattern of faster passage in the main study zone was also apparent among JBS-passed fish. The passage time ranged from 28.5 to 148.9 min during the day, but only 3.1–20.8 min at night. The mean of all of the passage time estimates for this area was 76.3 min during the day but only 7.2 min during the night.

To obtain more information on how fast these fish were passing through the forebays, the time it took to travel from 25 m away from the dam to the dam face was determined. In this location, the mean of all of the passage time estimates for turbine-passed fish was 70.4 min during the day but only 2.8 min during the night. Likewise, among JBS-passed fish, the mean of all estimates during the day was higher (80.1 min) during the day than at night (5.3 min).

## Discussion

Understanding the migratory behaviour of juvenile salmonids as they approach hydroturbines is critical to accurate understanding of the effects of turbine passage on overall dam passage survival and ensuring that these salmonid populations are conserved. Information about acclimation depth distributions could be used when releasing live, tagged fish into turbine intakes, allowing more accurate estimates of turbine-passage survival to be attained (Brown *et al*., 2014). Brown *et al*. (2014) provided a research framework for modelling barotrauma of fish exposed to rapid decompression. They found that the ratio of acclimation depth to nadir pressure is the most predictive variable for the probability of mortal injury of juvenile Chinook salmon. This framework includes the use of a combination of data on turbine exposure characteristics, including exposure data collected with Sensor Fish (Deng *et al*., 2007). Exposure data can be used in a laboratory setting to expose fish to realistic pressure and shear conditions. However, this framework is lacking input on the range of acclimation pressures for exposed fish. The present research provides a better understanding of the bounds within which we may expect fish to be acclimated.

One of the key assumptions about how these data can be applied to future modelling efforts is whether the fish monitored for this research were neutrally buoyant for most of the time they were in the dam forebays. This condition could be likely because fish use the physiological characteristics of their swim bladders to regulate their state of buoyancy and conserve energy. When fish are not neutrally buoyant, they have been observed to change their posture or swimming behaviour, resulting in greater energy use. For example, when Korsøen *et al*. (2010) made Atlantic cod (a physoclist with a closed swim bladder) positively buoyant by lifting the aquaculture cage in which they were held, their swimming speeds and tail beat frequencies increased by 1.5–4 times and 2–3 times, respectively. In addition, fish were swimming with their head down and tail up. In contrast, when Korsøen *et al*. (2010) made Atlantic cod negatively buoyant by lowering the aquaculture cage, fish were swimming with their head up and tail down. Atlantic cod increased swimming speeds 1.3–2.3 times and tail beat frequencies 1.4–2.3 times compared with their behaviour at neutral buoyancy. This is similar to how Pflugrath *et al*. (2012) described the behaviour of positively and negatively buoyant juvenile Chinook salmon. This increased energy use has led researchers to suggest that there are limitations in the depth that fish will move down and up in the water column (Mehner, 2012). For example, Eckmann (1991) suggested that whitefish (*Coregonus lavaretus*) would not increase their swim bladder volume more than 50% when ascending in the water column. Harden-Jones and Scholes (1981) suggested that, to reduce energy use, Atlantic cod (*Gadu morhua*) limit their vertical range to a 25% reduction in swim bladder volume and 50% increase in pressure. Although the information collected using acoustic telemetry technology for the present study on Snake River dams is revealing about the depth of migrating juvenile salmonids, research to confirm that they are neutrally buoyant at these depths would be useful.

This research indicates that the median depth at which juvenile salmonids are approaching turbines ranges from 2.8 to 12.2 m, with the depths varying by species/life history, year, location (which dam) and time of approach (i.e. day or night). Most (75%) of the fish entering turbines were typically approaching at depths ranging from 6.4 to 24.9 m. Fish passing through the turbines approached the powerhouse deeper than those passing the JBS. However, this is not surprising because the upper portions of the turbine intakes are screened to direct fish away from turbines and into bypass systems.

The comparison of acclimation depths and depth distributions between turbine-passed and JBS-passed fish in this study demonstrated consistently that turbine-passed fish were located deeper in the water column than JBS-passed fish. This result was consistent for all dams, species and years. Interestingly, these results were likely not to be the result of cues from the dam structure itself (e.g. flow), because acclimation depth calculations were limited to 3-D detections a minimum of 25 m away from the dam face, with data on fish within 25 m not being used for the present analyses. This suggests that individual characteristics, environmental conditions and/or operational conditions not investigated in this study may have influenced the ultimate route of passage. Future research is warranted to investigate variables that may be relevant for predicting the behaviour of fish prior to passage.

In this study, the effects of year, location and species on acclimation depths and depth distributions were investigated for fish that passed through the turbines and JBSs at LGS and LMN. We showed that fish tracked during 2012 were acclimated to shallower depths than those tracked during 2013. This result was similar for all fish that passed the dams and those fish that used the available turbine routes. On average, the summer (June and July) water temperature in 2013 was 2.3°C higher than in 2012 at the two dams. The average spill rate (water discharge) was 122–266 cubic metres per second higher in 2012 than in 2013. These two factors may have influenced the depth of travel for tagged fish. Salmonids have specific thermal tolerances, and it has been well documented that they will change their position in the water column to find an optimal temperature (Cunjak *et al*., 2005; Jonsson and Jonsson, 2009; Mehner, 2012; Dungale *et al*., 2013). Specifically, the higher temperatures observed in the Snake and Columbia rivers during 2013 may have resulted in fish migrating at deeper depths to stay within an optimal temperature range.

Location did not influence the acclimation depths and depth distributions of juvenile salmon examined in this study as much as other factors. For yearling Chinook salmon that passed through turbine routes during 2012 and for subyearling Chinook salmon that passed through the turbine routes during 2013, the acclimation depths were statistically different for fish that passed LGS and LMN. Despite the statistical significance, the medians differed by less than 1.8 m.

The species and life history of fish were shown to influence the acclimation depths and depth distributions observed in the present study. At both dams examined during 2012, subyearling Chinook salmon occupied the deepest median depths, compared with yearling Chinook salmon and steelhead. These results may be confounded by the differences in migration timing for these species. Yearling Chinook salmon and steelhead in the Columbia River basin begin migrating downstream in early spring each year, and the majority of the fish have left the system by mid-June. In comparison, subyearling Chinook salmon typically are not detected passing the main-stem dams in the Federal Columbia River Power System until mid-May, and individuals will continue to be collected at the juvenile fish facilities until October (although most fish will have passed by mid-July). Throughout the smolt out-migration period, the water temperature within the river system increases and typically peaks in late August. As a result of the differences in out-migration timing, subyearling Chinook salmon are generally exposed to warmer water temperatures than most yearling Chinook salmon and steelhead. This may partly explain the differences in acclimation depth observed. Given that subyearling Chinook salmon make their seaward migrations in warmer water temperatures, they may be seeking thermal refugia deeper in the water column. In addition, seasonal differences in predation (both avian and piscivorous fish) may influence the behaviour of these fish.

Among powerhouse-passed fish, there was an obvious difference in the depth distributions associated with the diel period. Steelhead and subyearling Chinook salmon passing powerhouses were consistently detected deeper in the water column during the night than during the day at both Snake River dams in both study years. In comparison, yearling Chinook salmon did not have large differences in depth distributions between diel periods. It is not clear what factors may have led to this behaviour. Vertical migrations are common among many fish species, in both lacustrine and marine environments [see Mehner (2012) for a recent review]. These movements or migrations are often associated with factors such as changes in light intensity over the diel period, feeding, predation or thermal variation; however, there is a paucity of information on vertical movements of juvenile salmon or other fish within rivers.

Within the Snake and Columbia rivers, fish have been expected to swim higher in the water column at night, when the likelihood of avian predation is lower. It is common for fish to move into deeper water during the day to avoid piscivorous fish. However, some assume that piscivores stop feeding at night, when there is less light (Mehner, 2012). There is not a clear indication why fish that passed powerhouses at night were located deeper in the water column than during the day. The water discharge across the dam was not operated in a distinct way during day and night.

It is unclear why the large difference in time to travel through the forebay and to the dam face occurred. It is possible that the fish may not be able to see or otherwise sense the face of the dam at night and may quickly move forward and into the entraining flow into the powerhouse. It may be possible that the fish can see the dam face during the day and take a longer time to search for a passage route. The pattern that fish move more quickly at night increased our understanding of survival and passage behaviour, which could be beneficial to estimate the impacts of the hydropower system on fish.

### Conclusion

This study successfully determined the depth distributions and estimated acclimation depths for juvenile salmonids that passed into the powerhouses (including turbines) at two Snake River dams. This project is unique because it used a large volume of data from a very large sample size of fish (approximately 28 000 fish of three species/life-history types), over multiple years and dams with sub-metre 3-D tracking accuracy. Multiple approaches were developed and evaluated for estimating the acclimation depth for individual fish prior to dam passage. Depth distributions of fish approaching the hydropower system before turbine passage were significantly influenced by factors of year, species/life history and diel period. This information has important applications to future turbine designs, operations and research for fish passage.

Additional analysis may help to explain further some of the results presented. Investigation of the influence of telemetry tags on the depth distributions of fish would allow better comparisons to run-of-the-river untagged populations. In addition, identification and understanding of the variables relevant for predicting the behaviour of fish prior to passage have management implications. Determining more detail about juvenile salmon behaviour, such as their orientation in the water column (e.g. migrating/moving head first or tail first) at different locations of their seaward migration would be valuable.

This research provides new and advanced understanding of depth distributions of juvenile salmonids on their seaward migrations based on a vast amount of high-resolution data. This valuable information will increase our understanding of existing data and help us to identify possible management operations or better turbine or overall dam designs. These improvements could play a major role in conserving seaward-migrating salmonids that interact with hydropower structures.

## Funding

This work was supported by the US Army Corps of Engineers, Walla Walla District.
